# Microplasma Treatment versus Negative Pressure Therapy for Promoting Wound Healing in Diabetic Mice

**DOI:** 10.3390/ijms221910266

**Published:** 2021-09-24

**Authors:** Pei-Lin Shao, Jiunn-Der Liao, Shun-Cheng Wu, Yu-Hsing Chen, Tak-Wah Wong

**Affiliations:** 1 Department of Nursing, Asia University, Taichung 41354, Taiwan; m8951016@gmail.com; 2 Engineered Materials for Biomedical Applications Laboratory, Department of Materials Science and Engineering, National Cheng Kung University, Tainan 70101, Taiwan; tony69896032000@hotmail.com; 3 International Center for Wound Repair and Regeneration, National Cheng Kung University, Tainan 70101, Taiwan; 4 Regenerative Medicine and Cell Therapy Research Center, Kaohsiung Medical University, Kaohsiung 807378, Taiwan; shunchengwu@gmail.com; 5 Department of Dermatology, National Cheng Kung University Hospital, College of Medicine, National Cheng Kung University, Tainan 704, Taiwan; 6 Department of Biochemistry and Molecular Biology, College of Medicine, Center of Applied Nanomedicine, National Cheng Kung University, Tainan 70101, Taiwan; Dr.kentwwong@gmail.com

**Keywords:** diabetic wound, nonthermal microplasma treatment, negative pressure wound therapy, re-epithelialization, transforming growth factor β signaling

## Abstract

The delayed healing response of diabetic wounds is a major challenge for treatment. Negative pressure wound therapy (NPWT) has been widely used to treat chronic wounds. However, it usually requires a long treatment time and results in directional growth of wound healing skin tissue. We investigated whether nonthermal microplasma (MP) treatment can promote the healing of skin wounds in diabetic mice. Splint excision wounds were created on diabetic mice, and various wound healing parameters were compared among MP treatment, NPWT, and control groups. Quantitative analysis of the re-epithelialization percentage by detecting Ki67 and DSG1 expression in the extending epidermal tongue (EET) of the wound area and the epidermal proliferation index (EPI) was subsequently performed. Both treatments promoted wound healing by enhancing wound closure kinetics and wound bed blood flow; this was confirmed through histological analysis and optical coherence tomography. Both treatments also increased Ki67 and DSG1 expression in the EET of the wound area and the EPI to enhance re-epithelialization. Increased Smad2/3/4 mRNA expression was observed in the epidermis layer of wounds, particularly after MP treatment. The results suggest that the Smad-dependent transforming growth factor β signaling contributes to the enhancement of re-epithelialization after MP treatment with an appropriate exposure time. Overall, a short-term MP treatment (applied for 30 s twice a day) demonstrated comparable or better efficacy to conventional NPWT (applied for 4 h once a day) in promoting wound healing in diabetic mice. Thus, MP treatment exhibits promise for treating diabetic wounds clinically.

## 1. Introduction

Diabetes has become a pandemic. According to the International Diabetes Federation (IDF), in 2019, approximately 463 million adults (20–79 years) had diabetes; this number is expected to increase to 700 million by 2045 [1]. Wound healing is a complex biological process that is considerably delayed in people with diabetes, and chronic nonhealing wounds are common [2]. In patients with diabetes, chronic skin wounds are difficult to heal and remain an unmet clinical need [3,4]. Therefore, wound healing in patients with diabetes is one of the most difficult problems for clinicians, and it is a heavy burden on patients, both physically and economically.

Over the past 20 years, negative pressure wound therapy (NPWT) has been used to effectively treat chronic wounds, and it can locally treat wounds of different sizes or complexities [5]. NPWT optimally controls the wound healing state. It involves applying negative pressure to induce mechanical stress in wound healing tissue and stimulate the mechanical conduction signaling pathway in connective tissue in order to guide tissue growth [6,7]. However, NPWT is cost- and time-intensive [7]. Another limitation is interference with granulation tissue formation. Granulation tissue formation is an essential event in early wound healing and provides a temporary scaffold for revascularization, cell attachment, and ingrowth [8,9,10]. In particular, diabetic wounds generally exhibit impaired granulocytic function [11]. In the preclinical model of NPWT, a longer treatment time, for example, 4 h a day for 2 consecutive days, only supports the production of granulation tissue response in the wound tissue [12,13]. Even if NPWT with a faster cycle time is used to prevent damage to nascent granulation tissue, a smaller amount of granulation tissue is formed [14].

Plasma medicine is a new field that combines nonthermal atmospheric pressure plasma physics with life sciences and medicine [15]. The innovative method of nonthermal microplasma (MP) treatment has multiple advantages, such as being contact-free, painless, and a nonallergic treatment and physical means of achieving an antibacterial effect [15]. The excited plasma, depending on the type of gas used, emits several reactive oxygen species (ROS) and reactive nitrogen species (RNS), such as ozone (O_3_), atomic oxygen (O), and nitric oxide (NO) [15]. By adjusting the composition of ROS and RNS produced by MP, MP treatment can be used for different biomedical applications, including sterilization, disinfection, and wound treatment [16,17,18,19]. An increasing number of exploratory preclinical and clinical studies have evaluated MP treatment for healing chronic diabetic wounds [20,21]. We previously developed a capillary tube-based MP device for treating burn wounds [18,22,23]. In our previous in vitro studies, we found that MP treatment enhances the proliferation and migration of fibroblasts, and this effect is achieved through stimulated fibroblast growth factor 7 (FGF7) release in fibroblasts [22]. We further found that MP treatment enhances wound healing in burn wounds in mice by enhanced angiogenesis and epithelialization processes [18]. In fractional CO_2_ laser-induced wounds, we also found enhanced wound healing after MP treatment [23]. Since MP treatment enhances wound healing in burn- and fractional CO_2_ laser-induced wounds in vivo, MP treatment may be used to enhance wound healing in a diabetic mouse. Moreover, the detailed molecular mechanism of how MP promotes wound healing remains rarely investigated.

In this study, we tested the hypothesis that MP treatment can enhance wound healing in a diabetic mouse model. A diabetic mouse model was designed to study the efficacy of MP treatment in diabetic wound healing and compare it with conventional NPWT. We also discuss the potential mechanisms underlying MP-induced wound healing.

## 2. Results and Discussion

### 2.1. Promoted Wound Closure of the Wound Bed

**Figure 1:**
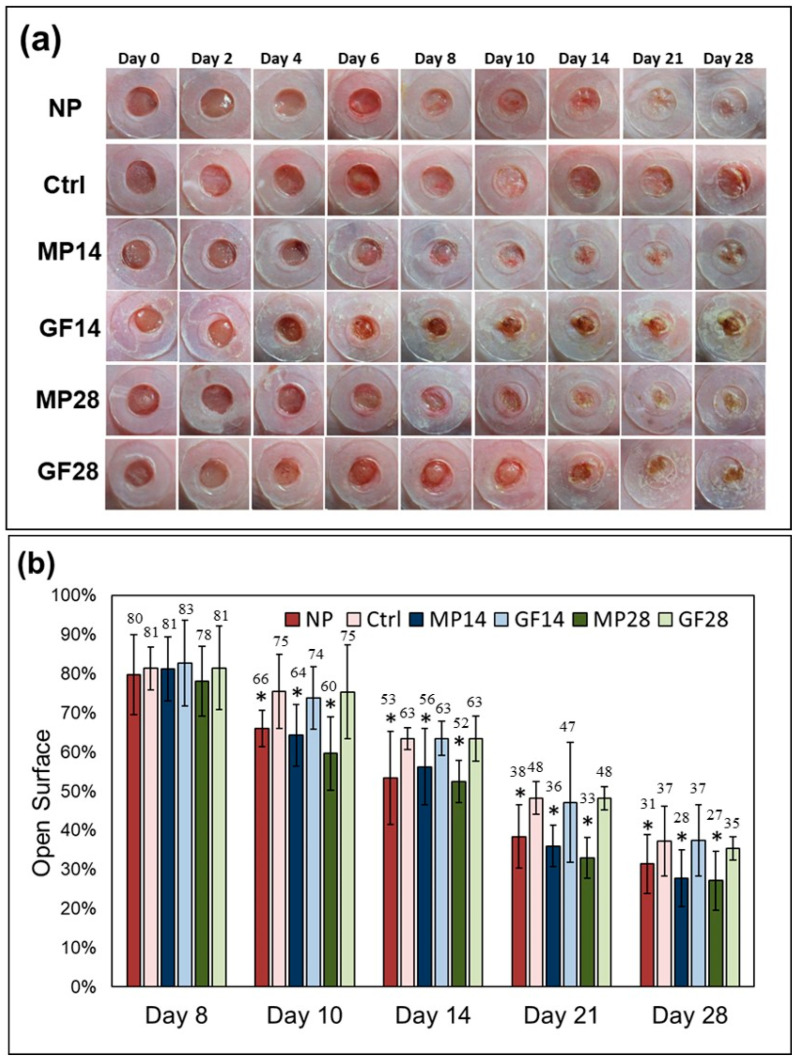
MP treatment promoted closure of the wound bed in the NP, control, MP14, GF14, MP28, and GF28 groups: (**a**) The splinted excisional wound in mice created on day 0 and measured on days 2, 4, 6, 8, 10, 14, 21, and 28 with representative photographs of the groups. (**b**) Measurements of open surface in mice conducted on days 8, 10, 14, 21, and 28. The values indicate the percentages of open surfaces in the groups. The values presented are the means ± SDs (*n* = 9), with * *p* < 0.05 compared with the control group.

To investigate the effects of MP treatment and NPWT on wound healing, the wound closure kinetics of the wound bed were investigated. The wound bed was measured at each indicated time point. Figure 1a presents representative photographs of the wound bed on days 0, 2, 4, 6, 8, 10, 14, 21, and 28 for the six groups. As summarized in Figure 1, no significant difference was found among the control, GF14, and GF28 groups; compared with the control group, the open surface of the wound bed in the NP, MP14, and MP28 groups was significantly reduced from days 10 to 28.

On day 14, the open surface of the wound bed in the NP, MP14, and MP28 groups decreased from 63% to 53%, 56%, and 52%, respectively. On day 21, the open surface on the wound bed in the NP, MP14, and MP28 groups further decreased from 48% to 38%, 36%, and 37%, respectively. Furthermore, on day 28, the open surface on the wound bed in the NP, MP14, and MP28 groups decreased from 37% to 31%, 28%, and 27%, respectively.

The quantitative results indicate significant reductions in the wound bed areas after MP treatment or NPWT, with no significant difference between the two. Thus, both MP treatment and NPWT may be competent in promoting wound closure of the wound bed.

### 2.2. Regrowth of New Tissues in the Wound Bed

**Figure 2:**
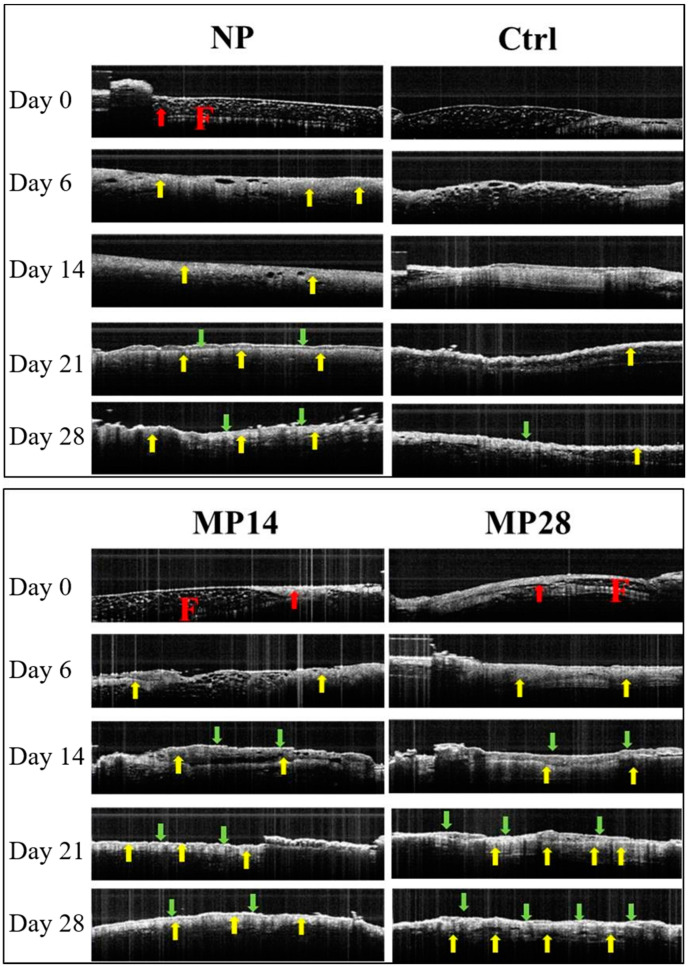
MP treatment promoted wound bed re-epithelialization on days 0, 6, 14, 21, and 28 (Study 1): Representative OCT images were acquired for the NP, control, MP 14, and MP 28 groups. “F” marked in red in the NP and MP14 groups indicates the tissue fluid; the red, yellow, and green arrows represent the muscle, dermis, and epidermis layers, respectively.

Figure 2 presents representative OCT images of the wound bed regions on days 0, 6, 14, 21, and 28 in the NP, control, MP14, and MP28 groups. The F marked in red and red arrows on day 0 indicate the fluid and the muscle layer, respectively, detected by OCT. The yellow and green arrows on days 6, 14, 21, and 28 represent the dermis and epidermis layers, respectively.

On day 0, no obvious changes were noted in the NP, control, MP14, and MP28 groups, except for a small amount of edema in the NP and MP28 groups. On day 6, new signals marked by yellow arrows were observed in the wound bed of the NP, MP14, and MP28 groups, but not in the control group. On day 14, obvious dermal and epidermal junctions, as indicated with the yellow and green arrows, were observed in the MP14 and MP28 groups, indicating the beginning of re-epithelization, whereas re-epithelization in the NP group started on day 21.

The re-epithelization process was much more intense in the MP28 group compared with that in the other groups. Overall, wound bed re-epithelialization was observed in the NP, MP14, and MP28 groups.

### 2.3. Re-Epithelialization by Measuring the Wound Gap in the Wound Bed

**Figure 3:**
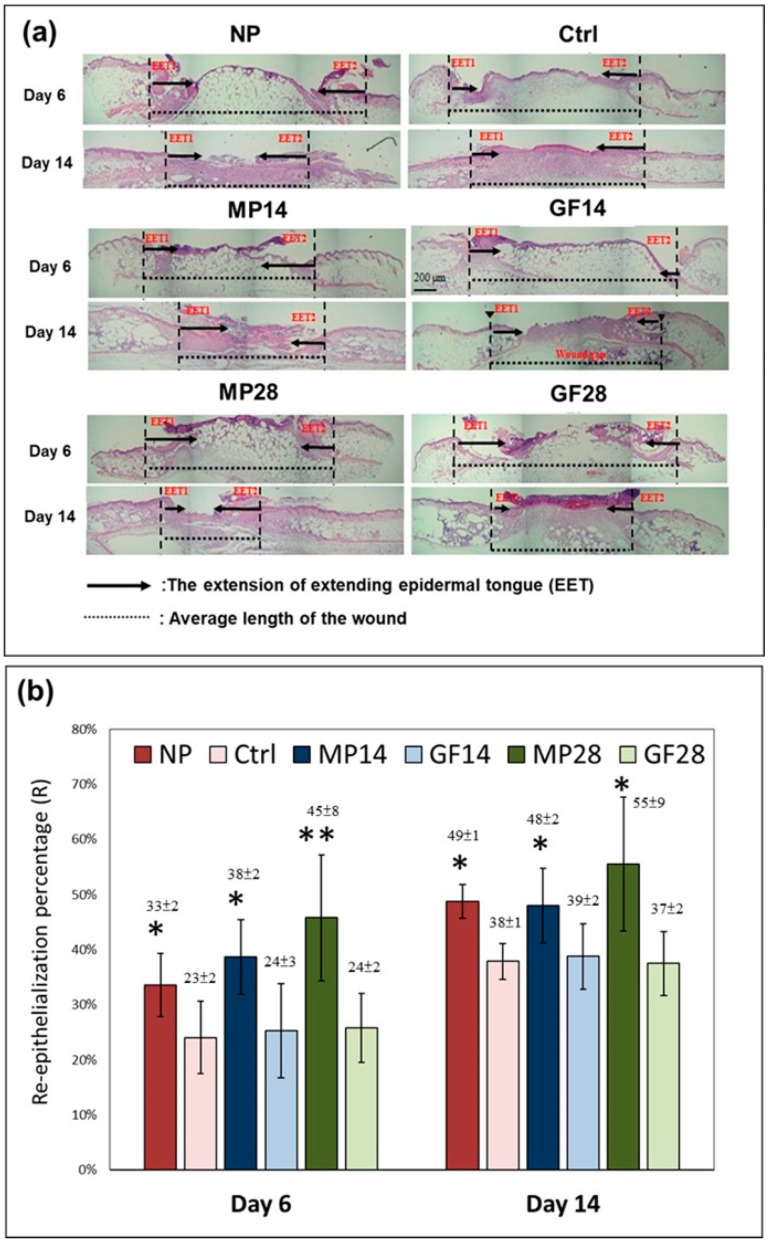
MP treatment promoted wound bed re-epithelialization of the NP, control, MP14, GF14, MP28, and GF28 groups on days 6 and 14 (Study 2). (**a**) Wound tissue sections stained with H&E with representative images. The distance between the two vertical dashed lines represents the average length of the wound (or wound gap), and, in between, the arrows denote the extension of the EET from the left (EET1) or right (EET2). (**b**) The quantitative data of the re-epithelialization percentage (R) in the wound gap. The values presented are the means ± SDs (*n* = 6), with * *p* < 0.05 and ** *p* < 0.01 compared with the control group.

Figure 3a presents the sections of the wound bed on days 6 and 14 in the NP, control, MP14, GF14, MP28, and GF28 groups stained with H&E. The histological evaluation of the re-epithelialization process was used to further confirm the results from the OCT images. The two vertical dashed lines represent the average length of the wound (or wound gap), and the arrows in between denote the extension of the EET from the left (EET1) or right (EET2). The distances between EET1 and EET2 were used to compare the extensions in the NP, MP14, and MP28 groups with the control, GF14, and GF28 groups, respectively.

The re-epithelialization percentage (R) was used to estimate the degree of re-epithelization; a value of 100% indicated complete wound bed re-epithelization. In Figure 3b, both MP treatment (MP14 and MP28 groups) and NPWT significantly increased the R of the wound bed on days 6 and 14 compared with the reference groups. The R values for the NP, MP14, and MP 28 groups were 34%, 39%, and 46%, respectively, on day 6 compared with 25% for the control, GF14, and GF28 groups. The R values for the NP, MP14, and MP 28 groups were 49%, 48%, and 56%, respectively, on day 14 compared with 38% for the control, GF14, and GF28 groups. On the basis of these results, we suggest that MP28 treatment can particularly enhance wound healing by promoting wound bed re-epithelialization.

### 2.4. Re-Epithelialization by Estimating the Formation of Cell Junctions in the Wound Bed

The expression of Ki67 and DSG1 in the EET of the wound bed was further analyzed to confirm the enhanced re-epithelization. Ki67 is a marker for proliferating keratinocytes, and the cell junction protein DSG1 is expressed mostly in the epidermis [24]. DSG1 is also an epidermal differentiation marker that maintains the structure of the epidermis through its adhesive function [25].

**Figure 4:**
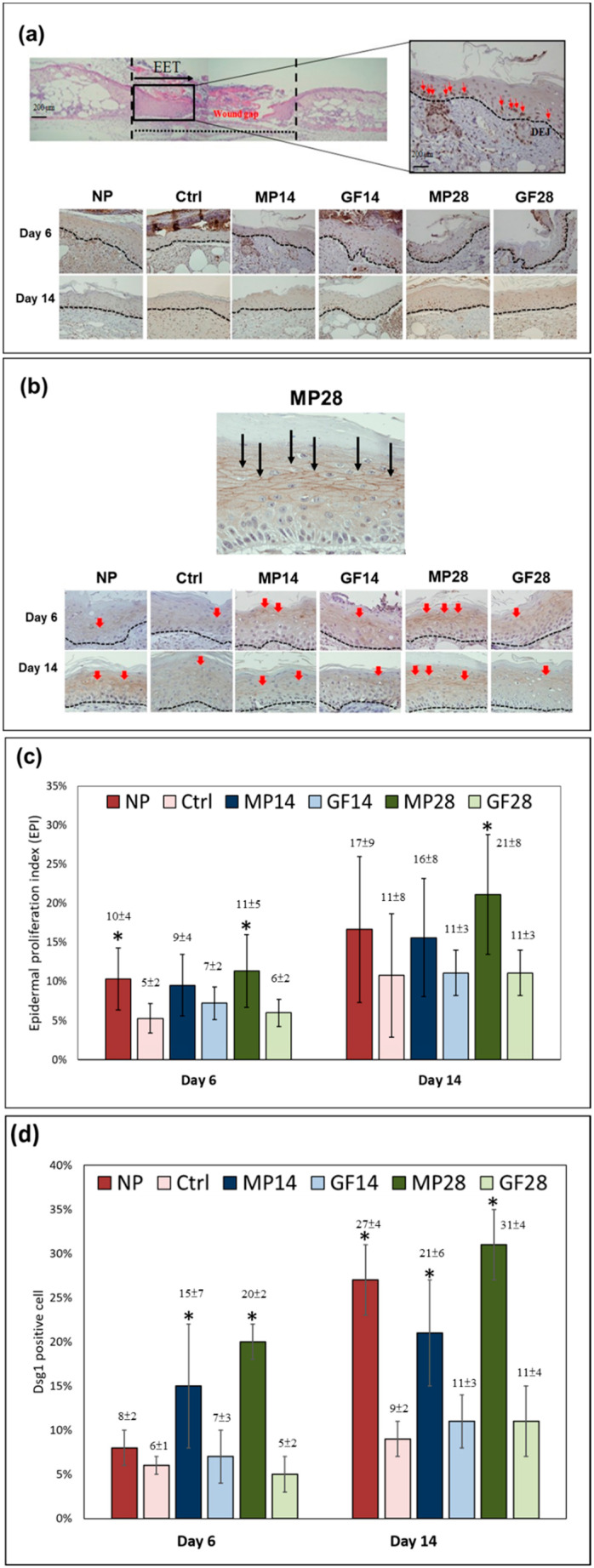
Immunohistochemical stain of Ki67 and DSG1 in the EET of the NP, control, MP14, GF14, MP28, and GF28 groups on days 6 and 14: (**a**) Representative images of Ki67 staining localized in the EET on the wound area (upper-left and lower images). Black curved dotted lines indicate the DEJ, and the red arrows indicate the stained Ki67 in keratinocytes around the DEJ (upper-right image). Scale bar = 100 μm. (**b**) Representative image of DSG1 staining localized in the EET of the wound area. Black arrows indicate the stained DSG1 in the EET (upper image). The IHC stain images of DSG1 represent the skin samples (lower images). (**c**) Graph of EPI. (**d**) Positive-stained DSG1 to evaluate the re-epithelization of the wound bed. The EPIs are expressed as the means ± SDs (*n* = 6), with * *p* < 0.05 compared with the control group.

In the upper-left image of Figure 4a, Ki67 staining is localized in the EET of the wound area. In the upper-right image, the black dotted lines represent the dermoepidermal junction (DEJ), with the red arrows indicating the stained Ki67 around the DEJ. The lower images of Figure 4a are the representative images of Ki67 staining localized in the EET of the wound areas of the NP, control, MP14, GF14, MP28, and GF28 groups on days 6 and 14. The lower images of Figure 4b are representative images of DSG1 staining of the groups on days 6 and 14. The red arrow indicates the brown, densely stained DSG1 signal. The brown DSG1 signal is positively stained around the cell body. The positive-stained cells show a cell-enhanced DSG1 signal and cell junctions. On day 6, the MP28 group had the brownest and most densely stained DSG1 signals, which represented the best cell junction. On day 14, the MP28 group still had a similar result. The NP and MP14 groups also displayed highly stained DSG1 signals that were less intense than that of the MP28 group. In the upper enlarged image of Figure 4b, black arrows indicate the stained DSG1 in the EET, which shows significant staining in the MP28 group on day 14, signifying the enhanced formation of cell junctions.

As illustrated in Figure 4a,b, both Ki67 and DSG1 were positively stained in the epidermis of the EET area. The epidermis in the EET of the wound bed shows increased Ki67-positive keratinocytes and more intense expression of DSG1. Among the six groups, more Ki67-stained keratinocytes (stained brown) were found in the NP and MP28 groups on day 6, whereas only Ki67-stained keratinocytes could be obviously found in the MP28 group on day 14. The DSG1 expression in the EET also shows that the MP28 group on day 14 had an enhanced formation of cell junctions.

As illustrated in Figure 4c, the EPI, as indicated in Equation (2), was also used to evaluate the re-epithelization of the wound bed of the NP, control, MP14, GF14, MP28, and GF28 groups on days 6 and 14 [24]. Compared with the control group, the NP group on day 6 and MP28 on days 6 and 14 significantly increased the EPIs that promote wound bed re-epithelialization. In Figure 4d, the positive-stained DSG1 was also used to evaluate the re-epithelization of the wound bed of the NP, control, MP14, GF14, MP28, and GF28 groups on days 6 and 14. Compared with the control group, the MP14 and MP28 groups on day 6 and NP, MP14, and MP28 on day 14 significantly increased the DSG1 positive-stained cells that promote wound bed re-epithelialization. This corresponds well with the results of Ki67 and DSG1 expressions in the MP28 group on days 6 and 14, signifying the enhancement of re-epithelialization by shortening the wound gap and forming a cell junction in the wound bed.

### 2.5. Promoted Enhancement of Wound Bed Blood Flow

**Figure 5:**
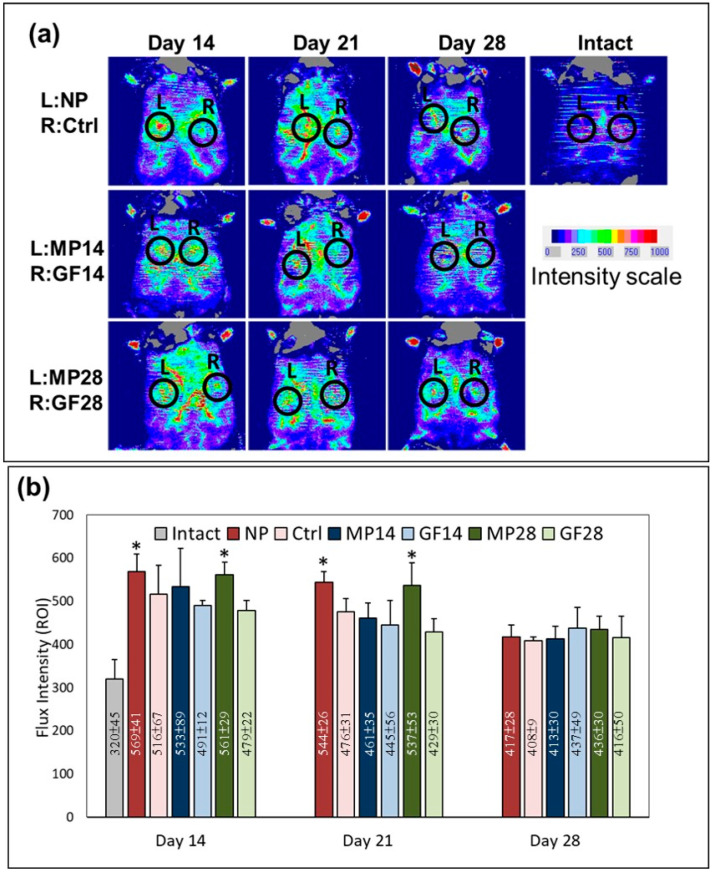
Assessment of blood flow at the wound as detected in laser Doppler scanning. (**a**) Representative blood flow images of GF14, MP14, GF28, MP28, control, and NP groups obtained on days 14, 21, and 28. Red and blue areas represent increased (normal) and reduced (or nonexistent) blood flow, respectively. (**b**) Quantitative data (**a**) showing blood flow in the ROI through flux intensity. Arbitrary units of flux intensity are expressed as the means ± SDs (*n* = 8), with * *p* < 0.05 compared with the control group.

In Figure 5a, the effects of wound bed blood flow in the NP, control, MP14, GF14, MP28, and GF28 groups on days 14, 21, and 28 are compared with the intact (wound) skin image. The red areas represent increased (normal) blood flow, and the blue areas represent reduced (or nonexistent) blood flow. The intact skin had a relatively low blood flow. On days 14 and 21, blood flow enhancement was observed in the NP and MP28 groups compared with the control group. As illustrated in Figure 5b, measurement of the blood flux intensity (ROI) revealed no significant difference between the NP and MP28 groups on days 14 and 21. The results thus indicate wound bed blood flow was increased in both groups.

### 2.6. Transforming Growth Factor β Signaling in the Epidermal Layer of Wound Tissues

Transforming growth factor β (TGFβ) signaling is critical for subsequent re-epithelialization and angiogenesis during wound healing and for successful wound closure [26]. The major intracellular mediators of TGFβ signaling are Smads, which are critical for TGFβ signaling. TGFβ mediates their signaling by binding to transmembrane TGFβ receptor II (TGFbRII). Activated TGFbRII binds and phosphorylates receptor-activated Smad2 or Smad3, which, upon heterodimerization with Smad4, translocates into the nucleus. Within the nucleus, activated Smad complexes become transcriptional factors [26].

**Figure 6:**
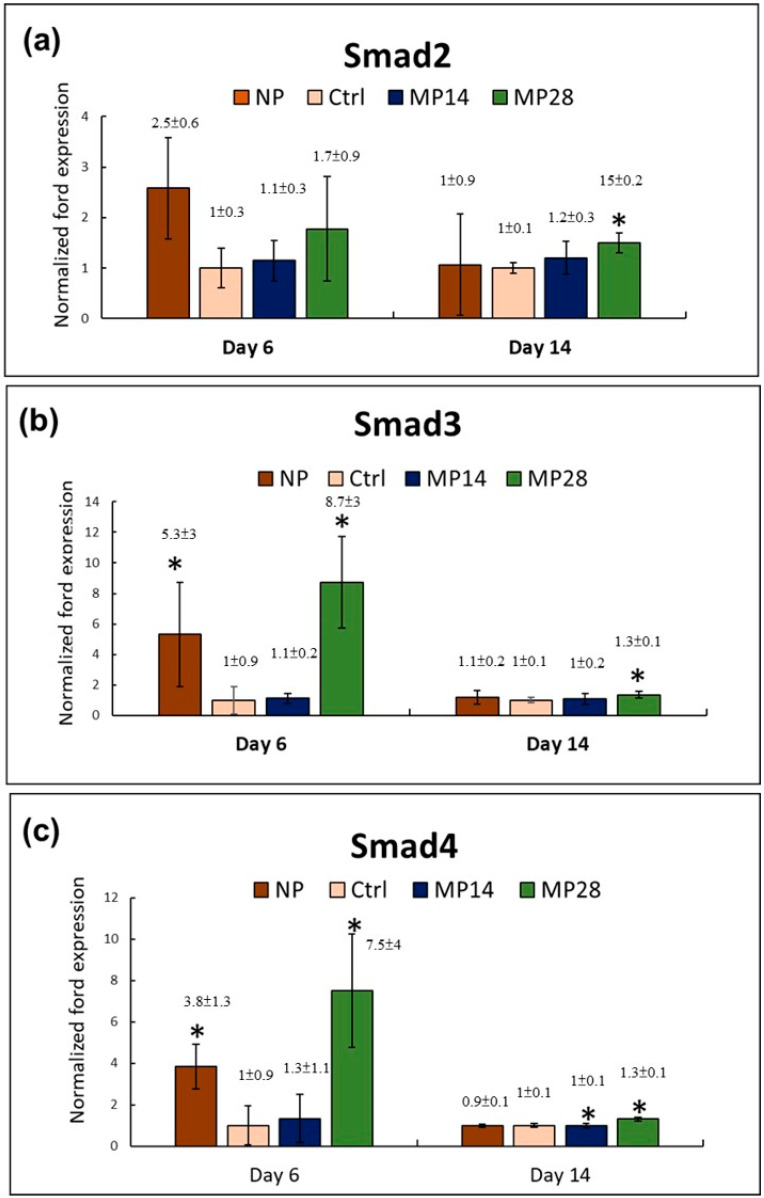
mRNA expressions of (**a**) Smad2, (**b**) Smad3, and (**c**) Smad4 in the epidermal layer in wound tissues of the control, MP14, MP28, and NP groups on days 6 and 14 after treatment. Gene expression levels are relative to those in the control group, which are defined as 1. The values presented are the means ± SDs (*n* = 6), with * *p* < 0.05 and compared with the control group.

As illustrated in Figure 6a–c, the mRNA expression of Smad2, 3, and 4 increased in the epidermal layer in wound tissues on days 6 and 14. The results demonstrate that both the NP and MP28 groups exhibited increased mRNA expressions of Smad3 and 4 in the epidermal layer in wound tissues on day 6. However, on day 14, the increased mRNA expressions of Smad2, 3, and 4 in the epidermal layer in wound tissues could only be found in the MP28 group. The results thus suggest that MP28 treatment induces increased expressions of Smad2, 3, and 4 in the epidermal layer in wound tissues.

### 2.7. Assessment of Wound Closure and Regrowth of New Tissues

MP treatment promoted wound healing in the diabetic mouse model, with a similar or better effect than NPWT in terms of wound closure, new tissue growth, re-epithelization, and wound bed blood flow. MP treatment can thus be considered an alternative approach to NPWT for treating diabetic wounds.

The mouse excisional wound healing model has been used extensively to study wound healing. However, contraction accounts for a large part of wound closure because mouse skin is mobile [27]. In this study, we used the mouse excisional wound splinting model. A splinting ring tightly adheres to the skin around the wound, preventing local skin contraction. The wound therefore heals through granulation and re-epithelialization similar to that in humans [27]. We observed that both MP treatment and NPWT exhibited similar effects on wound closure. However, the regrowth of new tissue under the wound bed area remains unclear.

OCT is a noninvasive cross-sectional imaging modality [28,29]. OCT was used for observing skin tissue in our previous study [23], and it can safely be used to differentiate various cutaneous structures [30,31]. The resolution enables the visualization of architectural changes of whole skin tissue in the wound bed area [32]. Accordingly, we used OCT to analyze the wound bed area after MP treatment and NP therapy. Our results reveal new tissue growth in the wound bed area after both treatments. This may be due to the proliferation of keratinocytes in the epidermal layer in these groups [23,30,31]. Compared with the control group, the MP treatment and NPWT groups displayed more homogenous architectural changes in the wound bed. Overall, our results suggest that both MP treatment and NPWT promote wound closure and wound bed tissue regrowth.

### 2.8. Assessment of Re-Epithelialization by Measuring the Wound Gap and Cell Junctions

Re-epithelialization plays a key role in wound closure [33]. To fully restore epidermal function at the wound site, epidermal regeneration is required through re-epithelialization, which involves keratinocyte proliferation, migration, and differentiation [34,35,36]. In the present study, both MP treatment and NPWT increased the re-epithelialization percentage (R) of the wound bed area from 29% to 26% for NP and 47% for the MP14 and MP28 groups.

Moreover, in the estimation of cell junction formation from IHC and the EPI, the increased Ki67-positive keratinocytes and more intense expression of DSG1 in the EET of the wound bed area were significant after MP treatment or NPWT. Analysis of Ki67 and DSG1 expression revealed that the MP28 group on days 6 and 14 had enhanced re-epithelialization in terms of a shortened wound gap and cell junction formation in the wound bed. The EPI results also indicate enhanced re-epithelialization.

### 2.9. Assessment of Blood Flow Recovery

Neoangiogenesis is an essential component of wound healing because new blood vessels supply cells at the wound site with nutrition and oxygen [37]. Diabetes decreases angiogenesis in healing wounds; therefore, diabetic wounds are characterized by decreased vascularity and capillary density [38].

In the present study, both MP treatment and NPWT enhanced wound bed blood flow, and no significant difference was found between the NP and MP28 groups on days 14 and 21. The results thus show that both the NP and MP28 treatments are competent in increasing wound bed blood flow.

### 2.10. Healing of Wound Tissue through NO Accumulation in Wound Tissue

NO regulates many human skin processes [39]. It is an essential regulator of both re-epithelialization and angiogenesis during wound healing [40]. Patients with diabetes have impaired re-epithelialization during wound healing [41]. Diabetic wounds have a microenvironment with elevated levels of glucose and ROS; high glucose levels reduce keratinocyte functions in vivo [36]. Diabetic wounds are also influenced by excess ROS production, which induces keratinocyte injury, dysfunction, and apoptosis [42] and enhances NO degradation [39,40].

However, diabetes also negatively affects wound healing by interfering with angiogenesis [2]. Low blood flow to the wound area is a major problem in diabetic wounds and can lead to impaired healing. Patients with diabetes often have a dysfunctional endothelium and insufficient recruitment of circulating endothelial progenitor cells, which are critical for wound repair [43,44,45,46]. The diabetic endothelium is also associated with enhanced NO degradation [40]. Although NO plays a vital role in re-epithelialization and angiogenesis in a diabetic wound, the direct employment of NO gas is limited by its high cost and potential toxicity at high NO concentrations [47].

By contrast, NO can be easily generated by nonthermal MP treatment directly and in the desired quantity at the site of use [15]. We have previously shown that MP treatment can increase NO accumulation in the tissue of a wound area without causing undesired heat-associated effects, which may damage the wound tissue [18,22,23]. As illustrated in Figure 4, Figure 5 and Figure 6, the increased wound blood flow and re-epithelialization in this diabetic mouse model were similar to our previous result in a laser-induced skin wound model [23]. On the basis of these reports, we suggest that MP treatment increases re-epithelialization and angiogenesis in the wound bed area of the diabetic wound mouse model; this result is most likely related to a slight increase in NO accumulation in the wound area.

### 2.11. Smad-Dependent TGFβ Signaling to the Enhancement of Re-Epithelialization

Keratinocyte migration and proliferation are crucial for successful re-epithelialization, and TGFβ signaling plays an important role in this process [26]. In contrast to normal wound healing processes that are characterized by activation of the TGFβ signaling, chronic nonhealing wounds have attenuated TGFβ signaling in the epidermis [26]. In other previous reports, MP treatment was indicated to increase TGFβ expression and enhance wound healing in streptozotocin (STZ)-induced diabetic rats [20], and it delayed the re-epithelialization of wounds when NO synthesis was inhibited [48]. NO synthesis in the epidermis of chronic wounds is shown to contribute to TGFβ signaling activation [26]. In this study, on day 14, the increased mRNA expressions of Smad 2, 3, and 4 in the epidermal layer in wound tissues could only be found in the MP28 group. Overall, these results suggest that Smad-dependent TGFβ signaling may contribute to the enhancement of re-epithelialization after MP treatment with an appropriate exposure time. However, how MP treatment regulates Smad-dependent TGFβ signaling during wound healing is still under study.

### 2.12. A Three-Dimensional In Vitro Model for Future Study

There are some points that need to be clarified. Firstly, although we found that MP treatment may promote chronic wound healing through NO accumulation, the therapeutic dose of NO accumulation needs to be clarified for future clinical applications. Secondly, the effects of MP treatment on the functions of fibroblasts and keratinocytes in chronic wound healing need to be further clarified. Fibroblasts and keratinocytes are two important cells that contribute to the wound healing process. Fibroblasts are an important cell type for the formation of granulation tissue [49]. Concurrently, keratinocytes proliferate and migrate toward the wound gap for the re-epithelialization process [49]. However, in a chronic wound, granulation tissue is diminished, re-epithelialization is halted, and the wound remains open [49]. Here, we found that MP treatment enhances wound healing in chronic wounds, accompanied by an increase in the expression of Smad 2/3/4 in the epidermis. TGFβ signaling plays a key role in keratinocyte epithelial-to-mesenchymal transition (EMT) [50]. TGFβ signaling is also involved in the differentiation of fibroblasts to myofibroblasts, which is characterized by the presence of alpha smooth muscle actin [51,52]. Myofibroblasts play a major role in the contraction and maturation of granulation tissue [52]. The direct cell–cell contact between fibroblasts and keratinocytes is important for TGFβ activation and initiation [53]. How MP treatment-induced Smad-dependent TGFβ signaling in keratinocytes and fibroblasts contributes to promoting chronic wound healing remains to be elucidated. Thirdly, the effects of MP treatment on enhanced angiogenesis in chronic wound healing need to be further clarified. It has been indicated that TGFβ signaling also regulates the angiogenesis process. For example, TGFβ is involved in angiogenesis by up-regulating vascular endothelial growth factor [26,54].

To answer these two questions, a simplified in vitro model needs to be developed. Considering the differences in skin architecture between mice and humans [55], two-dimensional monolayer culture may not capture the phenomenon of enhanced chronic wound healing after MP treatment in vivo. It is thus anticipated that a three-dimensional in vitro model that can simulate the in vivo microenvironment and the crosstalk between keratinocytes and fibroblasts, such as “human skin equivalents” [55], can be used for detailed molecular mechanism studies in the future.

## 3. Materials and Methods

### 3.1. Diabetic Mouse Model

All experiments were approved by the Institutional Animal Care and Use Committee (Approval No. 103067) of the Laboratory Animal Center of National Cheng Kung University (Tainan, Taiwan). We used db/db mice, which have significantly delayed healing, in our experiments [56]. Homozygous db/db mice have a genetic mutation in the leptin receptor, representing a model of type 2 diabetes mellitus characterized by hyperglycemia, obesity, hyperinsulinemia, and impaired wound healing [57]. Male diabetic mice (BKS.Cg-Dock7^m^ +/+ Lepr^db^/JNarl) were obtained from the National Laboratory Animal Center (NLAC, Taipei, Taiwan). This is a cogenic strain of db/db mice (BKS.Cg-Dock7^m^ +/+ Lepr^db^/J, #000642) from Jackson Laboratories (Bar Harbor, ME). NLAC mice become obese and hyperglycemic at approximately 5–6 weeks of age. All animals used in this study were 6–10 weeks old. Each mouse was housed in a cage with a 12 h light/dark cycle at room temperature and provided with standard food and water.

### 3.2. Splinted Excisional Wound Animal Model and Study Groups

An animal model of splinted excisional wound healing was created per established protocols, with minor modifications [27]. Briefly, the mice were anesthetized with 2.5% isoflurane at 1.5 L/min flow using a multicircuit anesthesia system (SAA2-3, Viking Medical, Minneapolis, MN, USA). Buprenorphine HCl (0.05 mg/kg, Buprenorphine HCl Injection, Ben Venue, OH, USA) and Lactated Ringer’s solution (10 mg/kg, Lactated Ringer’s injection USP, Baxter, Deerfield, IL, USA) were administered subcutaneously in a mouse following induction of anesthesia. The mouse was positioned in ventral recumbency on a temperature-controlled heating platform (ACT 100; World Precision Instruments, Sarasota, FL, USA). Hair on the dorsal surface of the skin was removed with an electric clipper, and the skin was sterilized with 4% chlorhexidine and 75% alcohol.

To create the splinted excisional wound model in mice, the locations of left and/or right wounds were marked on the dorsal surface with a surgical skin marker, and two full-thickness wounds were created at the designated location with an 8 mm sterile skin biopsy punch. Next, two donut-shaped splints (inner diameter: 10 mm, outer diameter: 14 mm) fabricated from a 1.6 mm-thick silicone sheet (Press-to-Seal Silicone Sheet JTR-S-2.0, Grace Bio-Labs, Bend, OR, USA) were prepared. The two donut-shaped splints were placed around the wound area and adhered with a tissue adhesive, which was a blend of (octyl/butyl) cyanoacrylate tissue adhesive (Gluture, Abbott Laboratories, North Chicago, IL, USA) and a glue (Krazy glue, Elmer’s product, Columbus, OH, USA). The splints around the wound were fixed using 6-0 nylon sutures (6-0 Ethilon Nylon Suture, Ethicon L.L.C., Cornelia, GA, USA). A semiocclusive adherent dressing (Tegaderm Film 9506 W, 3M Health Care, St. Paul, MN, USA) was applied to cover each wound.

### 3.3. MP Treatment and NPWT

We previously developed a capillary-based microplasma system that uses a controllable plasma composition and working temperature [19]. The following plasma excitation settings were controlled: a supply power of 17 W, 0.1% N_2_ added into Ar, and plasma plume-onto-wound temperature of 39 ± 0.5 °C [19]. The conditions of the nonthermal microplasma used in this study were based on our previous study—we found that MP treatment can effectively promote the wound healing of laser-induced mouse skin wounds [19]. In addition, the addition of 0.1% N_2_ to Ar microplasma can increase NO levels while reducing the heat-associated effects caused by plasma exposure. As the exposure time increased, the treatment site still remained within a biologically tolerable temperature range while accumulating NO in the wound tissue.

**Figure 7:**
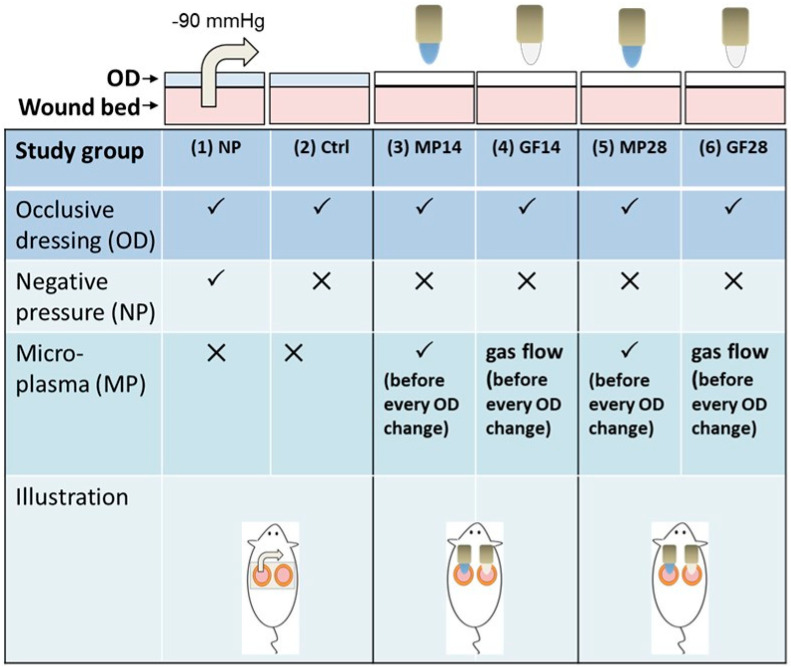
Schematic representation of the animal testing model with six study groups: (1) NP, (2) Control (Ctrl), (3) MP14, (4) GF14, (5) MP28, and (6) GF28. The operation sequences for MP treatment and negative NP therapy in mice are also illustrated.

For MP treatment in the wound area, the dressing was removed, and the wound area was then treated for 30 s. The diameter of the circled MP processing area was estimated to be 5 mm. For NPWT, the pressure was −90 mmHg, as per previous studies [58]. The mice with splinted excisional wounds were divided into the following study groups (Figure 7): (1) NP group: NPWT once per day for 4 h; (2) control (Ctrl) group: no treatment; (3) MP14 group: MP treatment once per day for 14 days (total 14 times); (4) GF14 group: gas flow (GF) treatment 14 times, corresponding to the MP14 group; (5) MP28 group: MP treatment twice a day at an 8 h interval for 14 days (total 28 times); (6) GF28 group: GF treatment twice a day at an 8 h interval for 14 days (total 28 times). The dressings in all groups were changed twice a day after treatment until the experiment ended.

The mice were placed under a warming lamp until they recovered fully from anesthesia. After MP treatment or NPWT, the mice were kept in cages in animal rooms with a 12 h light/dark cycle at room temperature (23–24 °C). The mice had free access to water and standard laboratory chow and were housed in separate cages to protect them from bites and to avoid fighting after wound creation. At the indicated time point, the mice were euthanized using overdose CO_2_ inhalation. The wound region skins of the mice in each group were harvested for further assessment.

### 3.4. Wound Closure Kinetics

For wound closure kinetics, the wound areas of mice in each group were compared using the following protocol [23]: Digital photographs of the wounds on each mouse captured on days 2, 4, 6, 8, 10, 14, 21, and 28 were compared with the initial photographs taken on day 0. On the basis of previous studies [23,59], the open surface of the wound area on day 0 was defined as 100%. The healing rates corresponding to the reduction in open surfaces were then measured. Digital planimetry software (ImageJ, National Institutes of Health, Bethesda, MD, USA) was used to quantify the area change in the wound bed. The investigators measuring the wounds were blinded to the groups and treatment conditions.

### 3.5. Noninvasive Assessment

For noninvasive assessment, the wound areas in each group were investigated as per protocol [59]. The images of the wound area were obtained through optical coherence tomography (OCT, OCS1300SS, Thorlabs, Newton, NJ, USA) along the transverse plane of the mice with dimensions of 6 × 6 × 3 mm^3^. Blood flow in the wound area was measured using a laser Doppler scanner (Moor LDIS, Moor, UK). For each measurement, a signal was generated that scaled linearly with tissue perfusion, defined as the product of the blood cell velocity and concentration. This signal was presented as a two-dimensional color image on a computer screen. The produced colors illustrated the spectrum of perfusion in the wound, where dark blue represented the lowest level of perfusion, and red represented the highest. The system simultaneously produced a photograph, allowing for a direct anatomical comparison of the corresponding areas of the wound. For each mouse, the region of interest (ROI) was selected after the image was imported into Moor LDI Image Review software (Version 5.3), and an area, including the wound sites (3 × 20 mm^2^), was scanned. The scans were performed on days 0, 6, 14, 21, and 28 after wound generation to assess blood flow at the wound sites.

### 3.6. Hematoxylin-Eosin Staining and Immunohistochemical Analysis

At the indicated time point, the mice in each group were euthanized; skin samples, including the entire wound and the 5 mm-wide surrounding margin of unwounded skin, were excised using a disposable microtome blade. Next, the skin samples were bisected through the center of the lesion to obtain the largest diameter of the wound margin and immediately fixed by immersion in 4% formaldehyde in phosphate buffer solution, followed by routine histological processing. The samples were serially sectioned at a thickness of 3 μm and then deparaffinized and rehydrated. The morphology of the wound area was stained using hematoxylin-eosin (H&E). For immunohistochemical (IHC) analysis, the sections were preincubated with a mouse/rabbit polydetector peroxidase block for blocking endogenous peroxidase activity (BioSB, Santa Barbara, CA, USA) and then incubated with primary antibodies against Ki67 (Abcam, Cambridge, MA, USA) and DSG1 (Abcam, Cambridge, MA, USA). The attached primary antibodies were detected with a biotinylated secondary antibody. Nuclei were stained with hematoxylin. Digital images were acquired using a digital camera (DFC450 C, Leica, Wetzlar, Germany) attached to a light microscope (DM IRB, Leica, Wetzlar, Germany). The images of Ki67- or DSG1-stained sections were quantified using Image-Pro Plus 5.0 software (Media Cybernetics, Silver Spring, MD, USA).

### 3.7. RNA Isolation and Quantitative Real-Time Polymerase Chain Reaction

At the indicated time points, the wound epidermis was peeled away from the underlying dermis as per the protocol [60]. The isolated epidermis from the wound area was frozen in liquid nitrogen and then homogenized using a tissue homogenizing kit (Omni Tissue Tip Homogenizing Kit, OMNI). The RNeasy Mini Kit (QIAGEN) was used to extract total RNA from these tissues according to the manufacturer’s instructions. Total RNA (1 μg per 25 μL reaction volume) was reverse transcribed into cDNA by using the M-MLV Kit (Promega Corporation, Madison, WI, USA). A real-time polymerase chain reaction (PCR) was performed using a GM SYBR qPCR Kit (GeneMark, Douala, Cameroon) in a quantitative real-time PCR detection system (ABI StepOne Real-Time PCR System; Applied Biosystems, Waltham, MA, USA). The cDNA samples (2 μL of samples in a total volume of 25 µL per reaction) were analyzed for the genes of interest. The primer sequences used to detect Smad2, 3, and 4 and glyceraldehyde-3-phosphate-dehydrogenase (GAPDH) are listed in Table 1. After real-time PCR, a dissociation (melting) curve was generated to determine the specificity of the reaction. The relative mRNA expression level of each target gene was calculated from the threshold cycle (Ct) value of each PCR product and normalized to the expression of GAPDH using the comparative Ct method [61]. For each gene of interest, readings were collected in each experimental group at every indicated time point.

### 3.8. Quantification of Re-Epithelialization

Wound bed re-epithelialization in each group was quantified as per established protocols [24], with minor modifications. The tissue sections of the wound area from each group were stained with H&E and photographed with a microscope. The average length of the wound (or length of wound gap) and the extension of the extending epidermal tongue (EET) were measured using the NanoZoomer virtual slide viewer software. The quantitative analyses of the re-epithelialization percentage (R) of the six groups were also evaluated with the use of the combined length of the extended EET (denoted as *l_EET1_* and *l_EET2_*) divided by the length of wounds gap (denoted as *l_wound gap_*) [24]. The value of R from the six groups was calculated using Equation (1):

(1) $$R=\frac{l_{EET1}+l_{EET2}}{l_{wound\;gap}}$$

### 3.9. Quantification of Proliferative Keratinocytes by Epidermal Proliferation Index

To analyze the proliferative keratinocytes in the EET of the wound area after NPWT or MP treatment, we quantified the epidermal proliferation index (EPI) as per established protocols [24], with minor modifications. The tissue sections of the wound area from each group were stained with Ki67 and DSG1 immunochemical staining to localize the proliferating keratinocytes in the epidermis of the EET. To calculate the EPI, the number of Ki67-expressing keratinocytes was divided by the total number of basal keratinocytes in the epidermis of the EET area to determine the percentage of proliferating cells as an indicator for proliferative activity, as shown in Equation (2). Furthermore, the total number of Ki67-positive keratinocytes was determined. The EPI of the six groups was also quantified as follows: the combined number of Ki67-positive keratinocytes in the epidermis of the EET (denoted as *n_EET1_* and *n_EET2_*) divided by the total number of basal keratinocytes in the epidermis of the EET (denoted as *n_total_*):

(2) $$EPI=\frac{n_{EET1}+n_{EET2}}{n_{total}}$$

### 3.10. Statistical Analysis

Data are expressed as the mean ± standard errors of the mean of the combined results of the experimental replicates. Statistical analyses were performed using SAS statistical software for Windows Version 8.2 (SAS Institute, Cary, NC, USA). Statistical significance was evaluated using Kruskal–Wallis analysis of variance, and multiple comparisons were performed using the post hoc Bonferroni-corrected Mann–Whitney U test. A value of *p* < 0.05 was considered to indicate significant differences.

## 4. Conclusions

Both MP treatment and NPWT stimulated wound healing in the diabetic mouse model. Both management methods enhanced wound closure, blood flow, and re-epithelialization. Moreover, the M28 group (30 s of therapy twice per day for 14 days) resulted in a similar or better therapeutic effect than that in the NP group (4 h of therapy once per day for 14 days). Thus, MP treatment, particularly in two short sessions per day, may be an alternative treatment method for promoting the healing of diabetic wounds.

## Figures and Tables

**Table 1:** Sequences of primers used in real-time polymerase chain reaction (Real-time PCR).

Gene	Forward Primer (5′-3′)	Reverse Primer (5′-3′)
GAPDH	TGCCCAGAACATCATCCCT	GGTCCTCAGTGTAGCCCAAG
Smad2	GTCAAGGGAAGGTGACCAGT	TGGCATAACCCAACACAGTT
Smad3	TGTGCGGCTCTACTACATCG	GCAGCAAATTCCTGGTTGTT
Smad4	CAGCCATAGTGAAGGACTGTTGC	CCTACTTCCAGTCCAGGTGGTA

## Data Availability

The data presented in this study are available on request from the corresponding author.
